# Effect of transcranial magnetic stimulation on prognosis in patients with postherpetic neuralgia and comorbid depression undergoing interventional neuromodulation therapy: protocol for a randomized double-blind placebo-controlled trial

**DOI:** 10.3389/fpsyt.2026.1744704

**Published:** 2026-04-10

**Authors:** Huichan Xu, Yanan Gao, Junpeng Yuan, Liqun Huang, Youjia Yu, Xiaohong Jin

**Affiliations:** 1 Department of Pain Medicine, The First Affiliated Hospital of Soochow University, Suzhou, Jiangsu, China; 2 Department of Pain Medicine, The Fourth Affiliated Hospital of Soochow University, Suzhou, Jiangsu, China; 3 Department of Pain Medicine, Suzhou Xiangcheng People’s Hospital, Suzhou, Jiangsu, China

**Keywords:** depression, PHN, postherpetic neuralgia, repetitive transcranial magnetic stimulation, rTMS

## Abstract

**Background:**

Postherpetic neuralgia (PHN) is often accompanied by depression, creating a vicious cycle that exacerbates symptoms and contributes to suboptimal treatment outcomes, even with interventional therapies. Repetitive transcranial magnetic stimulation (rTMS) has demonstrated potential in alleviating both pain and mood disturbances. However, its efficacy in enhancing prognosis when used alongside interventional neuromodulation therapy for PHN accompanied by depression remains inadequately explored and requires further investigation.

**Objective:**

This study aims to generate preliminary evidence on the efficacy and safety of rTMS in enhancing prognosis and alleviating pain in patients with PHN and mild to moderate depression undergoing interventional neuromodulation therapy.

**Methods:**

This study is a single-center, randomized, double-blind, placebo-controlled trial involving 174 adult patients with PHN. Participants will be randomly assigned, stratified by interventional neuromodulation therapy, to either the rTMS group (n=87) or the control group (n=87). Both groups will undergo either 10 Hz rTMS or sham stimulation for five consecutive days. The primary outcome is the incidence of poor prognosis at 3 months post-discharge. Secondary outcomes include the incidence of poor prognosis at 6 months post-discharge; Visual Analog Scale (VAS) sleep scores; short-form McGill Pain Questionnaire (SF-MPQ) scores; Self-Rating Depression Scale (SDS) scores; patient satisfaction; Pain Disability Index (PDI) scores; Multidimensional Fatigue Inventory-20 (MFI-20) scores; pregabalin oral doses; and the need for tramadol or antidepressants. Safety outcomes will include assessments of headache, pain at the stimulation site, neck pain, insomnia, muscle soreness, dizziness, nausea, tinnitus, irritability, tachycardia (heart rate > 100 bpm), and epilepsy. Data will be analyzed using a modified intention-to-treat approach.

**Discussion:**

This study aims to provide preliminary evidence on the efficacy and safety of 10 Hz rTMS in improving prognosis and alleviating pain in PHN patients with mild to moderate depression undergoing interventional pain management.

**Trial registration:**

https://www.chictr.org.cn/bin/project/edit?pid=261070, identifier ChiCTR2500096978.

## 1 Introduction

Postherpetic neuralgia (PHN) is one of the most common and debilitating complications of herpes zoster (HZ), significantly impairing patients’ quality of life (1, 2). In cases of refractory PHN, interventional neuromodulation therapies, including pulsed radiofrequency (PRF) and spinal cord stimulation (SCS), are widely employed and have demonstrated significant efficacy in pain relief (3). However, despite these treatments, up to 32.6% of patients experience poor outcomes, resulting in increased healthcare costs and even prolonged disability or impaired functional recovery (4). These poor outcomes typically manifest as persistent moderate to severe pain, impaired sleep quality, emotional distress requiring pharmacological intervention, or the need for repeated interventional treatments within a short timeframe following discharge (4, 5). Notably, a substantial proportion of PHN patients also suffer from depression, as chronic pain, inherently a persistent stressor, frequently induces depressive symptoms (5, 6). Studies indicate that up to 85% of chronic pain patients experience severe depression (7) and accumulating evidence suggests that pain and depression share common biological pathways and neurotransmitter systems, underscoring the importance of addressing both conditions concurrently (7). Moreover, patients with comorbid chronic pain and depression often have a worse prognosis than those with chronic pain alone. The bidirectional relationship between these conditions not only exacerbates symptom severity but also perpetuates a vicious cycle that complicates treatment outcomes (7, 8).

Repetitive transcranial magnetic stimulation (rTMS) is a non-invasive electrophysiological technique with significant potential for alleviating neuropathic pain by modulating cortical excitability and neurotransmitter expression (9). Research suggests that the primary motor cortex (M1) is the key target for rTMS in pain management due to its crucial role in pain modulation (10). Stimulation of the M1 region has been shown to attenuate pain signal transmission by influencing the thalamocortical pathway and central sensitization mechanisms (11, 12). Furthermore, high-frequency rTMS (10 Hz) has demonstrated potentially greater efficacy compared to lower frequencies in reducing pain, enhancing sleep quality, and improving overall well-being (13, 14). Given its efficacy in pain modulation, rTMS has also been explored as a therapeutic approach for various neuropathic pain conditions, including PHN, diabetic peripheral neuropathy, and trigeminal neuralgia (15). Moreover, existing studies have shown that rTMS has a therapeutic effect on negative emotions such as depression (16, 17). Building on these findings, we hypothesize that rTMS as an adjunct to interventional neuromodulation therapies could effectively reduce the incidence of poor postoperative prognosis in PHN patients with comorbid depression.

This study aims to evaluate the efficacy of perioperative rTMS compared to sham rTMS in PHN patients with comorbid depression undergoing interventional neuromodulation therapies. We hypothesize that rTMS will lower the incidence of poor prognosis and enhance postoperative pain outcomes in this patient population.

## 2 Methods

This study protocol was approved by the First Affiliated Hospital of Soochow University Ethics Committee (Approval No. 2025046). Written informed consent will be obtained from all participants prior to enrollment. All procedures will be conducted in accordance with the Declaration of Helsinki and Good Clinical Practice (GCP) guidelines. Additionally, this protocol is developed in accordance with the SPIRIT (Standard Protocol Items: Recommendations for Interventional Trials) guidelines, as outlined in **Supplementary File 1**.

### 2.1 Study design and patients

**Figure 1:**
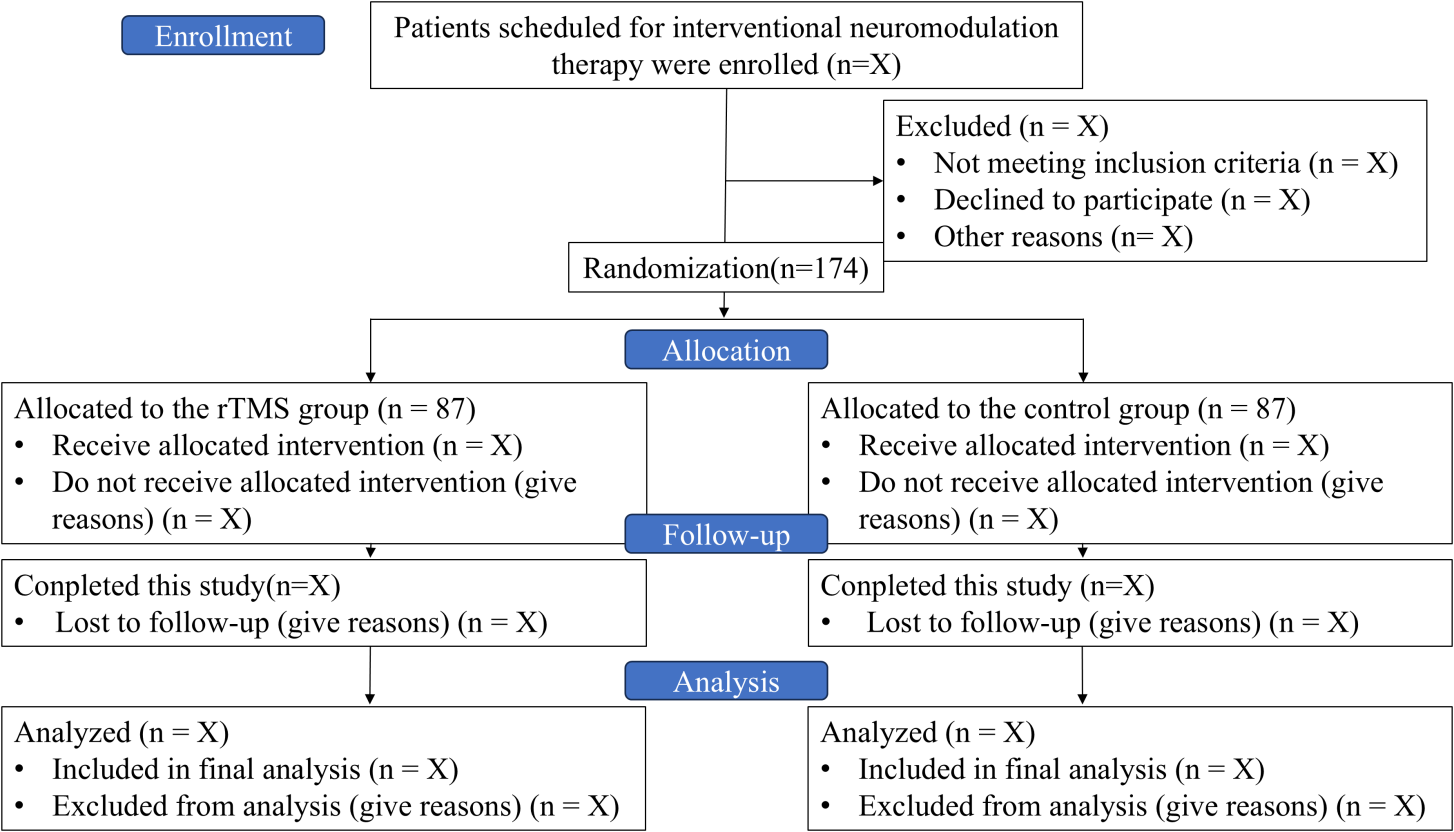
Study flow diagram showing the enrollment and randomization process.

This study is a single-center, prospective, randomized, patient- and assessor-blinded, parallel-group, controlled clinical trial. A total of 174 patients will be recruited from the First Affiliated Hospital of Soochow University and Suzhou Xiangcheng People’s Hospital. Initially, we planned to enroll and follow up patients from February 12, 2025 to December 31, 2025; data collection will be completed from January 1, 2026 to February 28, 2026; and results will be expected from March 1, 2026 to March 31, 2026. The study flow diagram is presented in **Figure 1**.

### 2.2 Inclusion criteria

Patients who meet the following criteria will be included:

- Diagnosed with unilateral PHN, defined as pain persisting for more than 1 month following clinical resolution of the rash (18, 19).
- Age 18 or older.
- Numeric Rating Scale (NRS) pain score ≥ 4, with a need for interventional neuromodulation therapy.
- Accompanied by mild to moderate depression (Self-Rating Depression Scale [SDS] score: 53-72, then confirmed by a psychiatrist).
- No evidence of aphasia or cognitive impairment.
- Scheduled for initial inpatient interventional pain management, including procedures such as PRF or SCS.
- Provided informed consent by the patient; family members will be informed as support persons.

### 2.3 Exclusion criteria

The exclusion criteria include:

- Severe systemic diseases, organic brain damage, or intracranial non-magnetic metal implants (including cochlear implants).
- History of mental disorders or use of psychiatric medications.
- History of implanted processors or implants (pacemakers, artificial heart valves) to vital signs in any part of the body.
- Severe cardiovascular, pulmonary, hepatic, or renal diseases.
- Coexisting pain conditions that may interfere with the assessment of PHN.
- Inability to communicate effectively to express subjective feelings.

### 2.4 Primary outcome

The primary outcome is the incidence of poor prognosis at 3 months post-discharge. Based on previous studies (4, 20), PHN patients will be classified as having a poor prognosis if they meet any of the following criteria: (1) moderate to severe pain, defined as a NRS score of 4 or higher on a 0-10 scale, where higher scores indicate greater pain severity; (2) pain-related sleep disturbances, defined as clinically observed or patient-reported difficulty falling asleep or frequent nighttime awakenings due to pain; or (3) pain-associated depression requiring pharmacological treatment, defined as a moderate or higher SDS score for which medication is indicated as recommended by a psychiatrist. Furthermore, patients who require a second or subsequent interventional pain management procedure within 3 months after discharge will also be classified as having a poor prognosis.

### 2.5 Secondary outcomes

**Figure 2:**
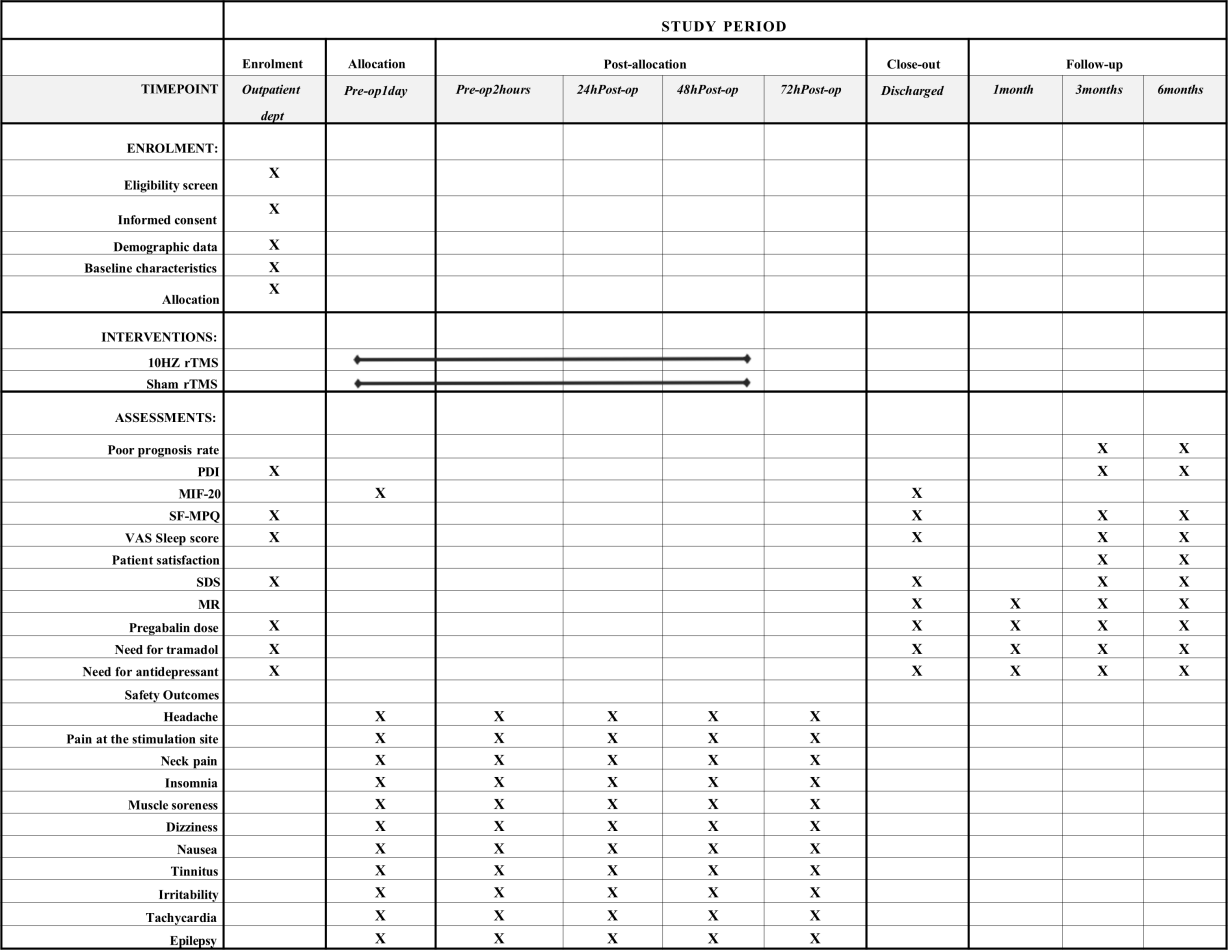
Study timeline and outcome measurement points across the 6-month period. rTMS, Repetitive Transcranial Magnetic Stimulation; PDI, Pain Disability Index; MIF-20, Multidimensional Fatigue Inventory-20; SDS, Self-Rating Depression Scale; SF-MPQ, the short-form of McGill pain questionnaire; Pre-op, Preoperative; Post-op, Postoperative; VAS, Visual Analogue Scale; SPIRIT, Standard Protocol Items: Recommendations for Interventional Trials.

Secondary outcomes will include the incidence of poor prognosis at 6 months post-discharge; VAS sleep scores; short-form McGill Pain Questionnaire (SF-MPQ) scores; SDS scores; patient satisfaction; Pain Disability Index (PDI) scores; Multidimensional Fatigue Inventory-20 (MFI-20) scores; pregabalin oral doses; and the need for tramadol or antidepressants. The key focus is on the trend changes in these indicators, and schedule of outcome assessment can refer to **Figure 2**.

### 2.6 Safety outcomes

Safety outcomes will include headaches, which are generally mild and can be alleviated by pausing treatment and resting. Other potential adverse effects include pain at the stimulation site, neck pain, insomnia, muscle soreness, dizziness, nausea, tinnitus, irritability, tachycardia (heart rate > 100 bpm), and epilepsy. Although patients with a history of epilepsy have been excluded from this study, epilepsy remains the most severe acute adverse reaction associated with rTMS, albeit with an extremely low incidence. In the rare event of a seizure, the trial for the individual will be immediately discontinued, the patient will be instructed to rest, and close monitoring will be conducted with symptomatic treatment as needed.

### 2.7 Randomization and blinding

An independent researcher will generate the randomization sequence using SPSS, implementing stratified randomization based on interventional neuromodulation therapy. The randomization list will be securely stored in sealed, opaque envelopes to ensure allocation concealment. Patients will be randomly assigned to either the rTMS group or the control group according to this sequence. To maintain blinding, patients, surgeons, outcome assessors, and statisticians will remain unaware of group assignments throughout the study.

### 2.8 Study interventions

In this study, patients will undergo 10 Hz rTMS or sham stimulation for five days, including two days before and three days after interventional pain management. Before treatment, the resting motor threshold (RMT) will be determined. Patients will be seated quietly in a treatment chair, and their hands will be cleansed with medical alcohol to remove oil. Electrode plates will then be attached to the designated locations: the ground electrode will be placed on the inner wrist, the recording electrode on the belly of the abductor pollicis brevis muscle, and the reference electrode on the tendon of the abductor pollicis brevis muscle. Using a positioning cap, the operator will apply single-pulse stimulation to the M1 region corresponding to hand movement on the affected side. The RMT will be determined as the stimulation intensity at which at least five out of ten stimuli elicit a wave amplitude exceeding 50 μV.

After setting the parameters, the treatment coil will be positioned tangentially to the scalp over the selected stimulation area, and the treatment session will begin. Patients will remain seated quietly, avoiding head movement, until the session concludes. The 10 Hz rTMS group will receive stimulation over the M1 region with the following parameters: frequency of 10 Hz, intensity at 80% RMT, 1,500 pulses per session, pulse duration of 0.5 seconds, inter-pulse interval of 3.0 seconds, and a total treatment duration of 17.5 minutes (21, 22).

For the control group, the same coil will be positioned over the M1 region, but the rTMS mode will be deactivated. To maintain blinding, pre-recorded rTMS treatment sounds from the active stimulation group will be played for the control group, ensuring a similar auditory experience. Patients will remain unaware of their group assignments to preserve the double-blind design. Treatments will be administered by medical professionals who are not involved in patient recruitment or data collection.

Patient blinding will be assessed via a post-treatment questionnaire asking participants to guess their treatment allocation.

To ensure treatment adherence, we have implemented the following strategies: (1) patient education on rTMS benefits at enrollment; (2) bedside administration of treatment by medical staff to minimize patient burden; (3) rescheduling of missed sessions within 24 hours.

These results about group assignments will only be revealed upon study completion. The schedule for patient enrollment, study interventions, and outcome assessments will adhere to the SPIRIT statement (**Figure 2**).

### 2.9 Data collection and monitoring

Patient information will be carefully reviewed by the research team using individual electronic medical records. Collected variables will include age, sex, BMI, disease duration, vaccination history, prior antiviral therapy, presence of prodromal pain, breakthrough pain, allodynia, hypertension, diabetes, malignancies, use of immunosuppressants, and the specific interventional treatment received (e.g., PRF or SCS). After discharge, data will be obtained through in-person or telephone follow-up and recorded in the follow-up management system. The duration of exposure will be defined as the number of years since the initial diagnosis of HZ. Significant life events-such as major illnesses, accidents, or bereavement-will also be documented.

To ensure data accuracy and integrity, all collected data will be double-entered independently by two trained research coordinators into an EDC system. Discrepancies will be resolved by consensus or consultation with the principal investigator. Regular monitoring visits and audits will be conducted by an independent data quality supervisor to verify source documents against entered data. Research staff involved in data collection will undergo standardized training in CRF completion and protocol adherence prior to trial initiation. A dedicated Data Monitoring Committee (DMC) will oversee overall data quality, compliance with Good Clinical Practice (GCP), and monitor protocol deviations.

Any serious adverse events (SAEs), regardless of their relation to the intervention, must be promptly reported to the principal investigator. The perioperative care team will take all necessary actions to ensure patient safety. These events must also be reported to the DMC within 24 hours for assessment and potential modification or discontinuation of the study protocol.

### 2.10 Sample size calculation

According to our earlier case-control study, the overall incidence of poor prognosis at 3 months following interventional pain management in PHN patients is approximately 32.6% (4). Since that publication, we have continued patient enrollment and follow-up over an extended period, and the observed incidence of poor prognosis at three months was 41.3%, which better reflects the current clinical characteristics and treatment patterns at our center. We hypothesize that rTMS may reduce this rate by 50% (i.e., to approximately 20.7%), and our sample size was calculated accordingly. Using a significance level (α) of 0.05 and a power (1-β) of 0.8, we estimate that 78 patients will be required per group under a 1:1 allocation ratio. Accounting for a potential dropout rate of 10%, the total sample size for this study will be 174 patients.

### 2.11 Statistical analysis

The normality of continuous variables will be evaluated using the Shapiro-Wilk test. Data following a normal distribution will be reported as mean (standard deviation, SD), while non-normally distributed data will be expressed as median (interquartile range, IQR). Group comparisons for normally distributed continuous variables will be conducted using independent t-tests or repeated measures ANOVA, as appropriate. For non-normally distributed variables, analyses will be performed using the Mann-Whitney U test or generalized estimating equations (GEE) to account for repeated measures and potential correlations. Categorical data will be presented as counts (percentages) and analyzed using the chi-square test or Fisher’s exact test, as appropriate, based on expected frequency distributions. The primary outcome, poor prognosis at 3 months post-discharge, will be analyzed using a logistic regression model, adjusting for the following pre-specified clinically relevant covariates: sex, age, type of interventional neuromodulation therapy (PRF vs. SCS), baseline NRS score, and baseline SDS score. Each component of this composite indicator will be subjected to exploratory analysis. Each secondary outcome will be analyzed according to its data type. Continuous outcomes (e.g., VAS sleep scores, SDS scores, PDI scores, etc.) will be analyzed using repeated measures ANOVA or GEE to assess changes over time and between groups. For categorical secondary outcomes (e.g., medication use, antidepressant requirement), comparisons will be made at each time point using chi-square or Fisher’s exact test, as appropriate. Safety outcomes will be analyzed at each time point separately using descriptive statistics and compared between groups using chi-square or Fisher’s exact test. No formal hypothesis testing or correction for multiple comparisons will be applied for safety endpoints, as these analyses are exploratory in nature.

Additionally, interactions between the treatment group and sex, age, type of interventional neuromodulation therapy (PRF vs. SCS), baseline NRS score, and baseline SDS score will be assessed. Multiple testing corrections for secondary outcomes will not be applied; therefore, these outcomes should be interpreted as exploratory findings. Odds ratios (OR) and corresponding 95% confidence intervals (CI) will be reported where appropriate. To assess the robustness of our primary findings, a sensitivity analysis will be performed. This sensitivity analysis will include only participants who fully adhered to the assigned intervention (i.e., completed all five rTMS or sham sessions) and completed the 3-month follow-up without major protocol deviations. The results will be compared to those from the modified intention-to-treat (mITT) analysis to assess the robustness of our primary outcome findings. The mITT will include all randomized participants who received at least one session of rTMS or sham stimulation.

Additionally, prespecified subgroup and interaction analyses were conducted according to age (< 65 vs ≥ 65 years), diabetes status (yes vs no), sex (male vs female), and type of interventional neuromodulation therapy (PRF vs SCS) to assess potential heterogeneity of treatment effects. Furthermore, interaction effects between the intervention and these subgroups on the primary outcome will be evaluated to explore potential effect modifications. All analyses will follow a modified intention-to-treat approach, including all randomized patients with available data. Statistical analyses will be performed using SPSS (version 25.0; IBM SPSS). Missing data will be imputed, and no interim analyses are planned. To minimize loss to follow-up and maintain an adequate sample size at the 3-month point, we have established a retention strategy that includes: regular reminders via phone calls and text messages, flexible follow-up scheduling, and engagement of family members when necessary. These measures are designed to ensure high participant retention throughout the study period.

### 2.12 Patient and public involvement

Neither patients nor members of the public will participate in the study’s design, recruitment, execution, or reporting. Study results will be shared with participants via email.

### 2.13 Unblinding principles and procedures

Planned unblinding: All participants will be unblinded following study completion, specifically after the final 6-month follow-up is concluded for all enrolled subjects.

Unblinding process: This will be managed by an independent DMC, which will maintain custody of all randomization records until the scheduled unblinding time point.

Emergency unblinding: In case of a serious adverse event or other critical incident during the trial, unblinding may be performed immediately to support appropriate medical care. The principal investigator will submit a formal request to the DMC, and the reason and process for unblinding will be fully documented.

## 3 Discussion

This randomized, double-blind, placebo-controlled trial involving 174 adult patients aims to evaluate the efficacy of 10 Hz rTMS in reducing post-procedural residual pain in PHN patients with comorbid depression undergoing interventional neuromodulation therapy. The primary objective is to assess the impact of rTMS on prognosis by determining the incidence of poor outcomes at 3 months post-discharge. Secondary objectives include evaluating the incidence of poor prognosis at 6 months post-discharge, as well as changes in VAS sleep scores, SF-MPQ scores, SDS scores, patient satisfaction, PDI scores, MFI-20 scores, pregabalin oral doses, and the need for tramadol or antidepressants. This study protocol was developed in accordance with the SPIRIT 2013 guidelines.

PHN is one of the most common and debilitating complications of HZ, significantly impacting patients’ quality of life. While pharmacological treatments remain the cornerstone of PHN management, patients with inadequate pain relief often require interventional techniques, such as PRF or SCS. Although these methods have demonstrated clear efficacy in pain control, they also have significant limitations. Even with these interventions, 18%-50% of patients continue to experience residual pain (1, 23, 24). The persistence of residual pain arises not only from central sensitization and abnormal neural circuits but also from neuroinflammation and maladaptive changes (25, 26). Therefore, the exploration of novel adjunctive treatments is critical.

In recent years, rTMS has gained attention for its potential in neuropathic pain management. Originally developed for psychiatric and neurological disorders such as depression and insomnia (27, 28), rTMS has since shown efficacy in chronic pain treatment, contributing to pain relief, improved sleep quality, and enhanced overall well-being (22). Notably, 10 Hz rTMS targeting the M1 has been effective in alleviating both persistent and episodic pain, including brachial plexus injury, diabetic peripheral neuropathy, and trigeminal neuralgia, while also mitigating pain-related anxiety and mood disturbances (29–31). Currently, researchers have shown rTMS is an effective mode in treating PHN, and 10 Hz rTMS is more effective than 5 Hz rTMS (32, 33). At the same time, stimulation of the M1 region showed a greater advantage than stimulation of the dorsolateral prefrontal cortex (DLPFC) region in patients with PHN (34). The M1 region serves as an optimal target due to its role in modulating thalamo-cortical pathways and attenuating central sensitization, mechanisms fundamental to neuropathic pain relief (35). Compared to alternative targets such as the DLPFC, M1 stimulation exhibits potentially greater efficacy and safety in pain management (10, 36). Therefore, this study will select 10 Hz rTMS to act on M1 region to explore the improvement of its prevention of poor prognosis.

rTMS exerts its analgesic effects through several mechanisms. First, rTMS modulates cortical excitability, improves cerebral blood flow and metabolism, induces adaptive plasticity, and attenuates neuroinflammation (27, 37–39). Furthermore, elevated inflammatory biomarkers and microglial activation observed in depression have been linked to depressive behaviors in animal studies (40–42). By targeting these shared neuroinflammatory and neuroplastic mechanisms, rTMS may simultaneously alleviate neuropathic pain and mood symptoms, creating a synergistic effect for PHN with comorbid depression.

The study employs a 5-day 10 Hz rTMS protocol targeting M1, an approach that demonstrates high therapeutic potential across various neuropathic pain conditions (22). While neuromodulation therapy such as SCS and PRF have advanced, these approaches primarily modulate pain signal transmission without directly addressing underlying nerve damage, maladaptive neuroplasticity, sustained central sensitization, or neuroinflammation (24, 43–48). In contrast, rTMS may facilitate neural repair and functional recovery by modulating cortical excitability and inducing neuroplastic adaptations (27, 38, 39), serving as a novel adjunctive strategy for PHN patients with persistent pain despite interventional treatment.

This study has several limitations. First, although the selected rTMS parameters are supported by existing literature, the optimal stimulation protocol for PHN remains uncertain and may require further refinement. Second, the 3-month follow-up period may not be sufficient to fully capture the long-term effects of rTMS, highlighting the need for future studies with extended follow-up durations. Furthermore, the study population may not encompass certain special cases, such as patients with long-term opioid use or severe mental health disorders, which could limit the generalizability of the findings to broader clinical settings.

In conclusion, this study aims to assess the efficacy of 10 Hz rTMS in reducing poor prognosis in PHN patients with comorbid depression. By conducting a rigorously designed randomized controlled trial, we seek to generate valuable insights into optimizing pain management strategies for PHN, ultimately improving postoperative outcomes and enhancing patients’ quality of life.
